# Synergy between Active Efflux and Outer Membrane Diffusion Defines Rules of Antibiotic Permeation into Gram-Negative Bacteria

**DOI:** 10.1128/mBio.01172-17

**Published:** 2017-10-31

**Authors:** Ganesh Krishnamoorthy, Inga V. Leus, Jon W. Weeks, David Wolloscheck, Valentin V. Rybenkov, Helen I. Zgurskaya

**Affiliations:** Department of Chemistry and Biochemistry, University of Oklahoma, Norman, Oklahoma, USA; Louis Stokes Veterans Affairs Medical Center

**Keywords:** *Acinetobacter*, *Burkholderia*, *Pseudomonas aeruginosa*, antibiotic resistance, multidrug efflux, outer membrane, permeability barrier

## Abstract

Gram-negative bacteria are notoriously resistant to antibiotics, but the extent of the resistance varies broadly between species. We report that in significant human pathogens *Acinetobacter baumannii*, *Pseudomonas aeruginosa*, and *Burkholderia* spp., the differences in antibiotic resistance are largely defined by their penetration into the cell. For all tested antibiotics, the intracellular penetration was determined by a synergistic relationship between active efflux and the permeability barrier. We found that the outer membrane (OM) and efflux pumps select compounds on the basis of distinct properties and together universally protect bacteria from structurally diverse antibiotics. On the basis of their interactions with the permeability barriers, antibiotics can be divided into four clusters that occupy defined physicochemical spaces. Our results suggest that rules of intracellular penetration are intrinsic to these clusters. The identified specificities in the permeability barriers should help in the designing of successful therapeutic strategies against antibiotic-resistant pathogens.

## IMPORTANCE

Multidrug-resistant strains of Gram-negative pathogens rapidly spread in clinics. Significant efforts worldwide are currently directed to finding the rules of permeation of antibiotics across two membrane envelopes of these bacteria. This study created the tools for analysis of and identified the major differences in antibacterial activities that distinguish the permeability barriers of *P. aeruginosa*, *A. baumannii*, *Burkholderia thailandensis*, and *B. cepacia*. We conclude that synergy between active efflux and the outer membrane barrier universally protects Gram-negative bacteria from antibiotics. We also found that the diversity of antibiotics affected by active efflux and outer membrane barriers is broader than previously thought and that antibiotics cluster according to specific biological determinants such as the requirement of specific porins in the OM, targeting of the OM, or specific recognition by efflux pumps. No universal rules of antibiotic permeation into Gram-negative bacteria apparently exist. Our results suggest that antibiotic clusters are defined by specific rules of permeation and that further studies could lead to their discovery.

## INTRODUCTION

In recent years, antibiotic-resistant Gram-negative bacterial species that have emerged in clinics have caused life-threatening infections that are effectively untreatable by antibiotic monotherapy (1, 2). These species are intrinsically resistant to antibiotics, albeit to various degrees, and gain further resistance when exposed to antibiotic treatments. The differences in antibiotic susceptibilities among Gram-negative bacteria are attributable to various factors, including the presence of chromosomally encoded enzymes that modify antibiotics, mutations, and variations in activities or amounts of antibiotic targets. Additionally, in all Gram-negative species, the low-permeability barrier of the outer membranes and multidrug efflux play key roles in resisting antibiotic challenges.

**FIG 1:**
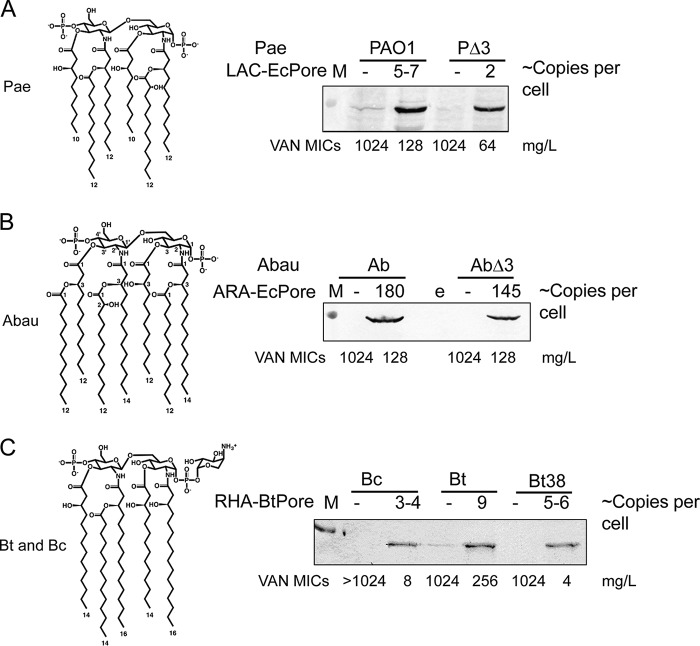
Structures of lipid A moieties and hyperporination of the outer membrane. The results of immunoblotting analyses performed with a monoclonal anti-His antibody, the copy number per cell of the expressed hyperpores, and MICs of vancomycin (VAN) in the induced cells are shown for *P. aeruginosa* PAO1 (Pae) (A), *A. baumannii* ATCC 17978 (Abau) (B), and *B. thailandensis* (Bt) and *B. cepacia* ATCC 25416 (Bc) (C). M, molecular marker lane.

The outer membrane (OM) of Gram-negative bacteria is an asymmetric bilayer composed of lipopolysaccharides (LPS) in the outer leaflet and glycerophospholipids (PL) in the inner leaflet (3). The major features of the LPS structure, such as the presence of lipid A, the core, and O-antigen chains, are conserved among various species while specific chemical structures vary broadly (Fig. 1). The different LPS aggregate into species-specific LPS-PL bilayers with various numbers of LPS molecules, thicknesses, surface charge distributions, and dynamics (4). These features, in turn, translate into differences in the permeability properties of LPS-PL bilayers (5–7). Most of the current knowledge on selectivity of the OM permeability barriers was determined on the basis of extensive studies of model organisms such as *Escherichia coli*. In enteric bacteria, antibacterial activities of large and polar antibiotics exceeding the size of general porins (>600 Da in *E. coli*) are usually the most restricted by OM, whereas the zwitterionic character in compounds correlates with increased permeation across the OM (3, 8–10).

The inner membrane (IM) is relatively permeable for the majority of amphiphilic drug molecules. However, it contains multidrug efflux pumps responsible for active nonspecific extrusion of toxic compounds from cells (11–13). Two types of efflux pumps operate and affect drug concentrations in different bacterial cell compartments (14). Some efflux transporters transport drugs across the IM and affect cytoplasmic drug accumulation (15, 16). Other transporters, such as those belonging to the resistance-nodulation-cell division (RND) superfamily, associate with additional proteins located in the periplasm and in the OM and function as transenvelope (across the two membranes) efflux pumps (17). These efflux pumps bind various substrates on the periplasmic side of the IM and translocate them across the OM into the external medium. Inactivation of transenvelope efflux increases bacterial susceptibility to various antibiotics, whereas their overexpression is a recognized cause of the clinical antibiotic resistance (18–20). The specificity of efflux pumps has been partially characterized in previous studies (21–23). Studies that included such Gram-negative bacteria as *Haemophilus influenzae*, *E. coli*, and *P. aeruginosa* revealed that the antibacterial activities of the very polar and low-molecular-weight (MW) compounds on the one hand, and of zwitterionic and high-MW compounds on the other, tend to be the least affected by efflux pumps, suggesting that such compounds are poor substrates for multidrug transporters (21, 22). In contrast, an increase in hydrophobicity was found to correlate with the increased propensity of a compound to be a substrate of efflux pumps in studies of *Salmonella enterica* serovar Typhimurium (23).

The exceptional efficiency of transenvelope efflux pumps is the result of a complex interplay between the two opposing fluxes of drugs across the two membranes. The experimental data and kinetic modeling data agree with the claim that Gram-negative bacterium cell envelopes serve to dramatically reduce the intracellular concentration of many antibiotics unless breached as a consequence of either efflux inactivation or an increase in the transmembrane flux (24–26). This synergistic character and the effectiveness of cell envelopes create major hurdles in the path of discovery and development of new therapeutics against Gram-negative pathogens (13, 27, 28). Significant efforts are presently directed at gaining a fundamental understanding of the permeability properties of the OM and at finding correlations between the physicochemical properties of compounds and their permeation across cell envelopes (1, 29). The task is complicated by the difficulties encountered in attempting the separation of the contributions of diffusion and active efflux in antibacterial activities (21, 22). Furthermore, heuristics established using model organisms, such as *E. coli*, tend to be poorly applicable to other Gram-negative bacteria and clinical isolates (30, 31).

We recently developed an approach that separates the contributions of active efflux and the passive barrier to activities of antibiotics and thereby sidesteps many of the aforementioned difficulties (25). In this approach, a modified *E. coli* FhuA siderophore uptake channel (EcPore) is expressed from the chromosome and used to create a large nonspecific pore in the OM of *E. coli*. EcPore is large enough for passage of small proteins (32) and does not discriminate between compounds on the basis of their hydrophilicity (25). In this study, we expanded this approach to analyze permeability barriers of four Gram-negative species, *P. aeruginosa*, *A. baumannii*, *B. cepacia*, and *B. thailandensis*. These bacteria were chosen because combating infections by such species represents an arduous task in clinics and because they differ significantly in their antibiotic susceptibilities, in the structure and composition of their OM, and in the activities and repertoires of the corresponding transenvelope efflux pumps. Thus, they appear to represent a diverse spectrum of Gram-negative cell envelopes. We constructed hyperporinated strains of these bacteria and analyzed the contributions of the OM permeability and intrinsic active efflux to the antibacterial activities of antibiotics belonging to different classes. The antibacterial activities were then correlated with permeability barriers that differed in chemical structures and properties. We unexpectedly found that mechanistic interplays between active efflux pumps and passive barriers universally protect bacteria from structurally diverse antibiotics, even those previously thought to be privileged in efflux avoidance. On the basis of their interactions with permeability barriers, antibiotics form four distinct clusters that occupy defined physicochemical spaces.

## RESULTS

### Hyperporination of the outer membrane is well tolerated by various species.

Contributions of the diffusion barrier and active efflux in antibacterial activities can be separated if a large nonspecific pore is inserted into the OM to allow unrestricted influx of antibiotics (25). Such a pore, however, must be properly folded and remain open in the OMs of various species. So far, only one effective modification of a pore has been constructed (25). To construct hyperporinated strains, the gene encoding the recombinant EcPore was integrated onto the chromosomes of *P. aeruginosa* PAO1, *A. baumannii* ATCC 17978, *B. thailandensis* E264 (Bt), and *B. cepacia* ATCC 25416 (see Table S1 in the supplemental material). As negative controls, empty expression cassettes were integrated into the respective strains as well. Here, the names of the strains comprise the strain abbreviation (Pa for *P. aeruginosa* PAO1, Abau [or Ab] for *A. baumannii* ATCC 17978, Bt for *B. thailandensis*, and Bc for *B. cepacia* ATCC 25416) followed by a designation representing the inducible promoter used for the induction (ARA for arabinose, LAC for IPTG [isopropyl-β-d-thiogalactopyranoside], and RHA for rhamnose) and the name of the pore, if present. Genetic tests, hypersusceptibility to the OM-impermeable antibiotic vancomycin, and immunoblotting analyses confirmed the successful integration into chromosomes and the inducer-dependent expression of the protein in PAO1-LAC-EcPore and Ab-ARA-EcPore (Fig. 1A and B). However, the protein failed to be expressed and function in Bt and *B. cepacia* ATCC 25416 strains.

10.1128/mBio.01172-17.6 TABLE S1  Strains and plasmids. Download TABLE S1, DOCX file, 0.02 MB. Copyright © 2017 Krishnamoorthy et al. 2017 Krishnamoorthy et al. This content is distributed under the terms of the Creative Commons Attribution 4.0 International license.

We next designed a pore (BtPore) using the OrbA siderophore uptake channel (33) from Bt as a template (see Text S1 in the supplemental material). To improve the expression, a synthetic gene encoding BtPore protein was cloned downstream of the rhamnose-inducible promoter (RHA). Genetic tests showed that the gene encoding BtPore was integrated into *glmS* sites present on the Bt (Bt-RHA-BtPore) and *B. cepacia* ATCC 25416 (Bc-RHA-BtPore) chromosomes. The protein was expressed in a rhamnose-dependent manner, was localized to the OM fractions, and enabled vancomycin-dependent killing of cells (Fig. 1C).

10.1128/mBio.01172-17.1 TEXT S1  Supplemental Methods. Download TEXT S1, DOCX file, 0.02 MB. Copyright © 2017 Krishnamoorthy et al. 2017 Krishnamoorthy et al. This content is distributed under the terms of the Creative Commons Attribution 4.0 International license.

The genes encoding EcPore and BtPore were further integrated onto the chromosomes of efflux-deficient variants of *P. aeruginosa* PAO1, *A. baumannii*, and Bt, correspondingly. PaΔ3-LAC and PaΔ3-LAC-EcPore cells are deficient in *mexAB*, *mexCD*, and *mexXY*, the inner membrane components of the major RND pumps of this species (34). BtΔ2-RHA and BtΔ2-RHA-BtPore strains lack the two RND-type pumps (AmrAB-OpmA and BpeAB-OpmB) that were previously reported to be constitutively expressed and to contribute to intrinsic antibiotic resistance in *Burkholderia* spp. (35). To analyze the role of efflux in *A. baumannii* ATCC 17978, we constructed isogenic strain AbΔ3 lacking AdeIJK, AdeAB, and AdeFGH, the three characterized RND efflux pumps of this species. For all constructed strains (Table S1), incubation of cells in the presence of respective inducers did not affect growth rates or cell densities, suggesting that hyperporination of all four species and their efflux-deficient variants does not result in significant growth defects (see Fig. S1 in the supplemental material). In agreement, the morphologies of hyperporinated cells and the protein contents of the inner and outer membranes were indistinguishable from those of the cells without pores (Fig. S1). Quantitative immunoblotting analyses (Fig. 1) showed that *P. aeruginosa* PAO1, Bt, and *B. cepacia* ATCC 25416 produce comparable amounts (2 to 10 copies per cell) of pore proteins in the OM. In contrast, the amounts of EcPore in *A. baumannii* ATCC 17978 cells were significantly higher at 150 to 200 copies per cell and were comparable to those previously reported in *E. coli* (25).

10.1128/mBio.01172-17.2 FIG S1  Morphologies, protein expression profiles, and growth curves of *A. baumannii* (A), *P. aeruginosa* (B), and *B. thailandensis* and *B. cepacia* (C). The strain numbering is the same that used in the protein expression profiles and growth curves. Bacterial growth was monitored by incubating bacteria in 96-well microplates at 37°C with shaking every 10 min and measuring optical density every 30 min. The shown growth curves represent averages of data from three independent experiments (error bars represent SD). Download FIG S1, TIF file, 2.5 MB. Copyright © 2017 Krishnamoorthy et al. 2017 Krishnamoorthy et al. This content is distributed under the terms of the Creative Commons Attribution 4.0 International license.

### Intracellular accumulation of fluorescent probes is defined by differences in permeability barriers.

Active efflux is effective because drugs are expelled across the low-permeability barrier of the OM (12, 17). Hence, increased influx across the OM could significantly diminish or even obliterate the contribution of active efflux. On the other hand, inactivation of efflux is expected to completely eliminate the permeability barrier in nongrowing cells, because the outer membrane slows but does not prevent the diffusion (3). To analyze the interplay between passive influx and active efflux in permeation of compounds and to establish whether hyperporination affects diverse species in the same manner, we measured the kinetics of intracellular accumulation of two fluorescent probes, Hoechst 33342 (HT) and N-phenyl-1-naphthylamine (NPN). The two probes are known substrates of bacterial efflux pumps (36–39) but have different physicochemical properties and intracellular binding sites. HT is an inhibitor of DNA topoisomerases (40), with MICs in the low-micromolar range in efflux-deficient strains (Table 1, Table 2, and Table 3). The fluorescence of water-soluble HT is significantly enhanced when the compound is bound to lipids and DNA (24, 41). NPN is a lipophilic, nontoxic compound and is fluorescent when bound to biological membranes (39).

**TABLE 1:** Susceptibilities to antibiotics of *P. aeruginosa* wild-type and efflux-deficient strains and their hyperporinated variants

Antibiotic	MIC (µg/ml)^a^			
	PAO1-LAC	PAO1-LAC-EcPore	PaΔ3-LAC	PaΔ3-LAC-EcPore
Bacitracin	>1,024	64	>1,024	64
Zeocin	512	32–64	16	2
Rifampin	16	0.5	16	0.5
Vancomycin	1,024	128	1,024	64
Polymyxin B	1.5	1.5	1.5	1.5
Nalidixic acid	64	32	8	2
Levofloxacin	0.125	0.063	0.031	0.004
Ciprofloxacin	0.063	0.031	0.016	0.004
Amikacin	2	2	1	1
Tobramycin + Mg^2+^	2	1	0.5	0.5
Azithromycin	128	4	4	0.063
Erythromycin	128	2	16	0.125
Tetracycline	4	0.5	2	0.125
Ampicillin	256	16	64	4
Carbenicillin	32	2	1	0.125
Cloxacillin	>2,048	512	128	8
Novobiocin	512	64	32	4
Chloramphenicol	8	2	1	0.125
Triclosan	1,024	1,024	32	8
HT (µm)	>64	64	16	2
Meropenem	0.5	0.25	0.125	0.03

^a^ Data represent results of at least three independent measurements and are expressed in micrograms per liter except as indicated.

**TABLE 2:** MICs of antibiotics in *B. thailandensis* wild-type and efflux-deficient strains and *B. cepacia* and their hyperporinated variants

Antibiotics	MIC (mg/liter)^a^					
	Bt-RHA	Bt-RHA- BtPore	BtΔ2-RHA	BtΔ2-RHA- BtPore	Bc-RHA	Bc-RHA- BtPore
Bacitracin	>1,024	1,024	1,024	8	>1,024	16
Zeocin	512	128	2	0.12	>1,024	128
Rifampin	16	8	4	0.03	8	0.06
Vancomycin	>1,024	256–512	>1,024	4–8	>1,024	8
Polymyxin B	>1,024	>1,024	>1,024	128	>1,024	512
Nalidixic acid	48	24	12	1.5	24	3
Levofloxacin	2	1	0.06	0.0156	2	0.06
Ciprofloxacin	1	0.5	0.06	0.016	1	0.03
Gentamicin	>64	>64	4	2	128	16
Amikacin	256	256	4	2	128	8
Kanamycin	>64	64	4	2	64	4–8
Tobramycin + Mg^2+^	64	64	1	1	32–64	4
Azithromycin	128	64	1	0.032	64	1–2
Erythromycin	512	256	1	0.5	256	8–16
Tetracycline	2	2	0.125	0.06	>8	8
Doxycycline	3	1.5	0.02	0.01	6	3
Ampicillin	256	256	256	4	>1,024	512
Carbenicillin	256–512	256	256	8	>1,024	128
Cloxacillin	256	128–256	32	0.5	256	4
Novobiocin	8	4	0.25	0.008	8	0.125
Chloramphenicol	8	8	4	1	16	4
Triclosan	120	30	0.12	0.06	>120	60
HT (µm)	>64	>64	1	0.5	>64	4–8
Meropenem	1	1	0.25	0.03	>4	0.125

^a^ Data represent results of at least three independent measurements and are expressed in micrograms per liter except as indicated.

**TABLE 3:** MICs of antibiotics in *A. baumannii* wild-type and efflux-deficient strains and their hyperporinated variants

Antibiotics	MIC (mg/liter)^a^			
	Ab-ARA	Ab-ARA-EcPore	AbΔ3-ARA	AbΔ3-ARA-EcPore
Bacitracin	256	32	512	64
Zeocin	8	0.5	1	0.125
Rifampin	2	0.5	1	0.25
Vancomycin	1,024	128	1,024	128
Polymyxin B	0.25	0.25	0.125	0.125
Nalidixic acid	3	1.5	1.5	0.75
Levofloxacin	0.125	0.125	0.031	0.031
Ciprofloxacin	0.063	0.008	0.008	0.004
Gentamicin	4	4	8	8
Amikacin	8	4	4	4
Tobramycin + Mg^2+^	4	2	4	2
Azithromycin	0.31	0.04	0.31	0.08
Erythromycin	5	1.25	1.25	0.08
Tetracycline	0.125	0.031	0.008	0.004
Doxycycline	0.04	0.02	0.0016	0.0016
Ampicillin	2,048	128	1,024	128
Carbenicillin	2,048	32	2,048	64
Cloxacillin	1,024	128	128	16
Novobiocin	3.1	1.56	0.025	0.025
Chloramphenicol	32	8	8	4
Triclosan	0.125	0.063	0.002	0.001
HT (µm)	25	3.125	0.78	0.025
Meropenem	0.25	0.063	0.125	0.063

^a^ Data represent results of at least three independent measurements and are expressed in micrograms per liter except as indicated.

**FIG 2:**
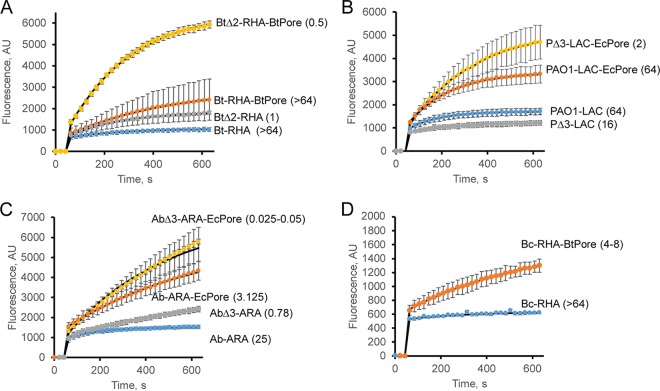
Intracellular uptake of the DNA-binding drug HT in different species and their hyperporinated and efflux-deficient derivatives. Data represent real-time kinetics of HT (6 μM final concentration) uptake into *B. thailandensis* (A), *P. aeruginosa* PAO1 (B), *A. baumannii* ATCC 17978 (C), and *B. cepacia* ATCC 25416 (D). In all panels, blue represents parent strains, orange represents hyperporinated parent strains, gray represents efflux-deficient mutants, and yellow represents efflux-deficient hyperporinated strains. Values in parentheses represent MICs (in milligrams per liter). Error bars represent standard deviations (SD) (*n* = 3). AU, arbitrary units.

In these experiments, induced cells were suspended in buffered glucose solution supplemented with either HT or NPN. In all four species, the overall results with respect to HT accumulation kinetics were similar (Fig. 2). Following the initial burst, caused by binding to cell membranes, HT fluorescence steadily increased, reflecting the cytoplasmic accumulation of DNA-bound HT (Fig. 2). However, the species differed significantly in the rates and intracellular accumulation levels of HT. The initial binding of HT to membranes in the Bt-RHA and Bc-RHA cells was at least 2 to 3 times lower than in the *P. aeruginosa* PAO1 and *A. baumannii* ATCC 17978 cells, suggesting that cell envelopes of these species are significantly less permeable to HT (Fig. 2A and D). In all four species, hyperporination increased the influx across the OM by at least 2 to 4 times, as seen from the increased intracellular accumulation of HT in the strains producing the pores (Fig. 2). The pore-mediated increase in HT accumulation was the largest in PAO1-LAC-EcPore and Ab-ARA-EcPore cells and resulted in a corresponding 4-to-8-fold drop of HT MICs in these cells (Fig. 2B and C). The increases in the accumulation of HT were lower and similar in Bt-RHA-BtPore and Bc-RHA-BtPore, respectively, but only hyperporination of *B. cepacia* ATCC 25416 resulted in a measurable, >4-fold decrease in the MIC. Thus, the total intracellular HT concentration, as measured by changes in HT fluorescence, and the concentration of external HT needed to inhibit the target, as measured by MICs, relate to each other in a complex, indirect manner.

Inactivation of efflux led to a lower (up to 2-fold) increase of intracellular HT accumulation in Bt and *A. baumannii* ATCC 17978, whereas PΔ3-LAC cells accumulated HT at levels lower than those seen in the parent PAO1-LAC strain. Thus, inactivation of efflux and hyperporination have different effects on the penetration of HT across the cell envelope. However, in all species, efflux-deficient hyperporinated cells accumulated the largest amounts of HT and were the most susceptible to the antibacterial activity of HT, with HT MICs at 25 to 50 nM in AbΔ3-ARA-EcPore and 0.5 and 2.0 μM in BtΔ2-RHA-BtPore and PΔ3-LAC-EcPore, respectively.

Thus, in all species, active efflux remained functional in hyperporinated cells and reduced the intracellular accumulation of HT despite increased influx of the probe across the outer membrane. At the same time, inactivation of efflux reduced the permeability barrier only partially, and the slow uptake across the outer membrane defined the rates of penetration. The changes in the rates of uptake caused by hyperporination and efflux inactivation often exceed the sum of changes caused by each of the two factors alone (24). For example, efflux inactivation in the BtΔ2-RHA strain produced a 2.1-fold change in the rate of HT uptake and hyperporination alone in Bt-RHA-BtPore produced a 1.4-fold change, whereas the BtΔ2-RHA-BtPore strain accumulated HT 10 times faster than Bt-RHA (Fig. 2). Similarly dramatic changes could also be seen in the MICs of HT and other antibiotics in all species (Table 1, Table 2, and Table 3). These results reveal that efflux inactivation and hyperporination act in synergy with each other. Indeed, should hyperporination and efflux inactivation be independent, then the changes in the rates of uptake and in the drug susceptibilities resulting from the combination of the two effects should equal the product of the increases caused by each factor alone.

**FIG 3:**
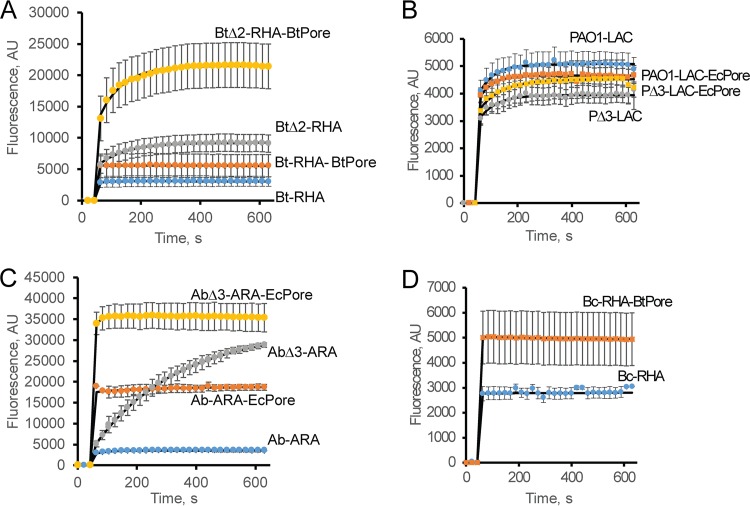
Intracellular uptake of the lipophilic probe NPN in *A. baumannii* ATCC 17978 and *B. thailandensis* and their hyperporinated and efflux-deficient derivatives. Data represent real-time kinetics of NPN (6 μM final concentration) uptake in Bt cells (A), *P. aeruginosa* PAO1 (B), *A. baumannii* ATCC 17978 (C), and *B. cepacia* ATCC 25416 (D). In all panels, blue represents parent strains, orange represents hyperporinated parent strains, gray represents efflux-deficient mutants, and yellow represents efflux-deficient hyperporinated strains.

The results described above also show that the effect of hyperporination and efflux inactivation on HT accumulation is species specific, as can also be seen in the accumulation of NPN, a membrane-binding dye (Fig. 3). Hyperporination increased the accumulation of NPN in Bt and *B. cepacia* ATCC 25416 membranes only modestly (by ~2 times) (Fig. 3A and D) but resulted in a large (more than 6-fold) increase in NPN levels in *A. baumannii* ATCC 17978 membranes (Fig. 3C). On the other hand, no significant changes in NPN levels could be seen in hyperporinated PAO1-LAC-EcPore cells (Fig. 3B). Unlike with HT, the loss of active efflux resulted in higher levels of NPN accumulation in Bt and *A. baumannii* ATCC 17978 cells than in hyperporinated cells (Fig. 3A and C), suggesting that the outer membranes of these species are more permeable to this probe and that efflux plays the dominant role. In particular, the outer membrane of efflux-deficient AbΔ3-ARA did not impose a significant permeability barrier for NPN as seen by the continuous accumulation of this probe inside the cells over the time course of the experiment. Surprisingly, the NPN levels in the efflux-deficient hyperporinated PΔ3-LAC-EcPore cells were only slightly higher than in PAO1-LAC cells. This result suggests that in *P. aeruginosa* PAO1 cells, this hydrophobic probe binds to the OM and does not reach the inner membrane during the time course of these experiments.

Taken together, these results show that the four Gram-negative species are protected by permeability barriers that differ significantly in their properties. Either hyperporination or efflux inactivation leads to species-specific changes in the kinetics of intracellular accumulation of fluorescent probes. The interplay between active efflux and the permeability barrier of the OM generates strong synergistic effects.

### Synergism of active efflux and passive uptake in antibacterial activities.

The results described above show that hyperporination and efflux inactivation have different effects on compound accumulation and that these effects are also specific to bacterial species. We next analyzed the interplay between active efflux and transmembrane diffusion in antibacterial activities of compounds with widely diverse physicochemical properties and mechanisms of action. We found that the antibacterial activities of all tested antibiotics were affected in a species-specific manner by efflux inactivation or hyperporination or both (Table 1, Table 2, and Table 3 and Fig. S2).

10.1128/mBio.01172-17.3 FIG S2  Contributions of the outer membrane barrier and active efflux to susceptibilities of bacteria to antibiotics. (A) The effect of the OM hyperporination on susceptibilities of different species in the presence and absence of major efflux pumps expressed as fold change in MICs (OM ratios). (B) The effect of inactivation of efflux on susceptibilities of *A. baumannii* ATCC 17978, *P. aeruginosa*, and Bt to antibiotics expressed as fold change in MICs (Efflux ratios). FQ, fluoroquinolones; AG, aminoglycosides. Download FIG S2, TIF file, 1.9 MB. Copyright © 2017 Krishnamoorthy et al. 2017 Krishnamoorthy et al. This content is distributed under the terms of the Creative Commons Attribution 4.0 International license.

**FIG 4:**
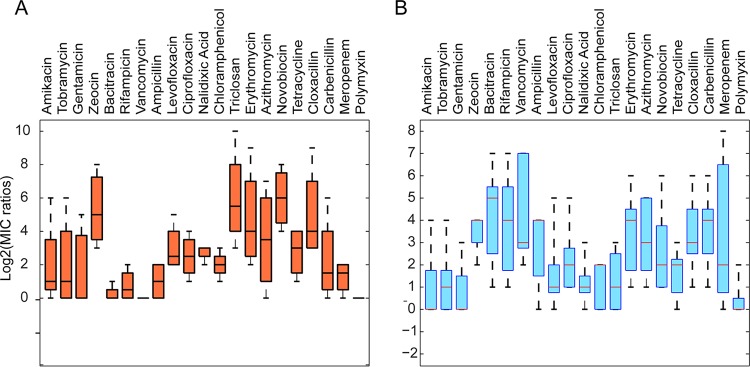
Contributions of the outer membrane barrier and active efflux to susceptibilities of bacteria to antibiotics. (A) A box plot of efflux ratios calculated from drug MICs measured in *A. baumannii* ATCC 17978, *P. aeruginosa* PAO1, *E. coli*, and Bt. Data represent merged ratios of (i) wild-type drug MICs to the drug MICs determined for efflux-deficient strains (i.e., PAO1/PΔ3) and (ii) the drug MICs determined for hyperporinated efflux-plus strains to the drug MICs determined for hyperporinated efflux-minus strains (PAO1-Pore/PΔ3-Pore). The median and the middle half of the distributions of ratios are shown as boxes. (B) Data were determined as described for panel A but for OM ratios calculated from drug MICs measured in *A. baumannii* ATCC 17978, *P. aeruginosa* PAO1, *E. coli*, *B. cepacia* ATCC 25416, and Bt. Data represent merged ratios of (i) wild-type drug MICs to the drug MICs determined for the hyperporinated strains (i.e., PAO1/PAO1-Pore) and (ii) drug MICs determined for efflux-deficient strains to drug MICs determined for hyperporinated efflux-deficient strains (i.e., PΔ3/PΔ3-Pore).

We next calculated the drug MIC ratios of parental cells and either efflux-deficient or hyperporinated cells, as appropriate. Grouped according to the effects of efflux inactivation (efflux ratios) and hyperporination (OM ratios), the MIC ratios displayed broad species- and antibiotic-dependent variations (Fig. 4). Notably, there was no obvious correlation between the consequences of transporter inactivation and those of hyperporination, indicating that the two effects are largely independent of each other. For example, in all species, fluoroquinolones, chloramphenicol, polymyxin, and antibiotics with a MW of >800 were affected by efflux only weakly, as seen from narrow distributions of respective efflux ratios. In contrast, the OM ratios varied broadly for most antibiotics, with zeocin and polymyxin showing the narrowest distributions.

**FIG 5:**
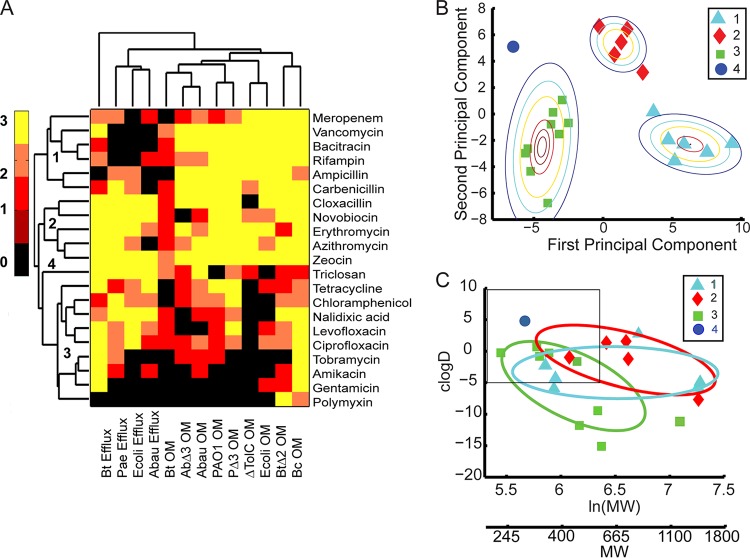
Statistical analyses of relationships between efflux and OM ratios in different species. (A) Hierarchical clusters and the heat map of all measured efflux and OM ratios. Data represent efflux ratios (wild-type strain [WT]/efflux mutant) with native membranes and OM ratios (parent-parent-Pore) for efflux-proficient (Bt, *A. baumannii* ATCC 17978, PAO1, and *E. coli*) and efflux-deficient (AbΔ3, PΔ3, ΔTolC, and BtΔ2) cells. The heat scale is the base-2 logarithm of the change in MICs. The identified clusters of antibiotics are numbered. (B) Principal-component analysis of antibiotic clusters with best-fit Gaussian distributions. Clusters are numbered as described for panel A. (C) Sizes [log(MW)] and hydrophobicities (logD) of antibiotics belonging to the indicated clusters and fitted to the Gaussian distributions as indicated.

To reveal potentially hidden features in the accumulated data, we performed cluster analysis of the measured ratios. These analyses also included the MIC ratios determined previously in efflux-deficient and hyperporinated *E. coli* strains (25). Strikingly, the tested antibiotics separated into several groups, which could be seen both in linkage analysis (Fig. 5A) and in principal-component (PC) decomposition (Fig. 5B). Since clusterization is based on biological recognition, each group includes structurally diverse antibiotics belonging to different classes. The distinction between the groups was mainly based on the magnitude of the effects caused by efflux inactivation (second principal component [PC2]) and hyperporination (PC1) (Fig. 5B and Table S3). Notably, the two effects were well separated from each other on the dendrogram (Fig. 5A), further pointing to their lack of interdependence. On the lower level, the effects of hyperporination on efflux-deficient and -proficient cells clustered according to species. Hence, the distinction between effects of the pore on MICs appears to be driven by biological determinants.

The four clusters of antibiotics revealed by distance analysis were as follows. Group 1 comprises antibiotics that are strongly potentiated by hyperporination but not efflux inactivation in all species (with the exception of Bt, which is discussed below). This group includes vancomycin, bacitracin, and rifampin as well as some beta-lactams (Fig. 5A). Vancomycin and bacitracin are large-molecule antibiotics with a MW of >1,400 that target peptidoglycan synthesis but cannot permeate the OM. Rifampin is a transcriptional inhibitor which is large (MW of 850) and hydrophobic. Beta-lactams are relatively hydrophilic and permeate the OM through porins. Thus, to cross the OM, all these antibiotics must use porins or channels, which could be general or specific for different drugs, and their activities are expected to be defined by biological determinants. Indeed, the OM ratios of the group 1 antibiotics differ significantly between the species. For example, hyperporination significantly (>32-fold) potentiated the activity of meropenem in *B. cepacia* ATCC 25416 but had a modest (2-fold to 4-fold) potentiation effect on other species (Fig. S2). This result suggests that *B. cepacia* ATCC 25416 lacks a specific OprD-like porin that facilitates the uptake of carbapenems in *P. aeruginosa* PAO1 and *A. baumannii* ATCC 17978 (42, 43).

Group 2 includes antibiotics, the antibacterial activities of which are strongly affected by both efflux inactivation and hyperporination. The group 2 antibiotics are the macrolides erythromycin and azithromycin, novobiocin, cloxacillin, and zeocin (Fig. 5A). The MICs of these antibiotics differ broadly between the species, reflecting species-specific differences in efflux capacities and OM selectivity (Table 1, Table 2, and Table 3). For example, *P. aeruginosa* PAO1 is resistant to novobiocin, with a MIC of 512 mg/liter, which contrasts with the relative susceptibilities of *A. baumannii* ATCC 17978 (MIC, 3.1 mg/liter), *B. cepacia* ATCC 25416 (MIC, 8 mg/liter), and Bt (MIC, 8 mg/liter). Hyperporination resulted in only a modest 2-fold potentiation of novobiocin activity in *A. baumannii* ATCC 17978 and Bt cells, but significant (16-fold to 32-fold) potentiation was found in *P. aeruginosa* PAO1 and *B. cepacia* ATCC 25416 cells. The efflux ratios of novobiocin were high in the *A. baumannii* ATCC 17978, *P. aeruginosa* PAO1, and Bt species. Surprisingly, the antibacterial activity of zeocin, a glycopeptide antibiotic with a mass of 1,428 Da, was strongly affected by active efflux in all species (Table 1, Table 2, and Table 3). The synergistic effect of inactivation of efflux pumps and hyperporination resulted in staggering 64-fold, 256-fold, and 4,096-fold changes in the MIC of zeocin in AbΔ3-ARA-Pore, PaeΔ3-LAC-Pore, and BtΔ2-RHA-Pore, respectively (Table 1, Table 2, and Table 3 and Fig. S2).

Group 3 antibiotics were modestly affected by efflux or porination. This group comprises fluoroquinolones, tetracycline, and chloramphenicol as well as polycationic aminoglycosides and polymyxin. These antibiotics have low efflux and OM ratios either because they are able to penetrate permeability barriers of all species by avoiding efflux and diffusing across the OMs or because they target the OM itself by binding and disintegrating the OMs in susceptible species (44–46). We found accordingly that inactivation of efflux and hyperporination of OM (separately or in combination) affected the bacterial susceptibilities to these antibiotics only weakly (Table 1, Table 2, and Table 3).

Finally, group 4 is represented by only one antibiotic, triclosan, which uniquely showed a strong effect of efflux inactivation in all tested bacteria and a moderate effect of hyperporination. In general, a single point could not be called a cluster. However, triclosan (i) is well outside the other three clusters, (ii) displays a unique pattern of behavior, and (iii) is the furthest away from the rest of antibiotics on the clustering dendrogram. Analyses of a wider range of compounds will likely identify other members of this group. Thus, the found clusters produced clearly discernible patterns with respect to effects of efflux inactivation and hyperporination.

Clusterization results based on, separately, the effects of the pore (Fig. S3A and B) or efflux (Fig. S3C and D) bore little resemblance to each other, in further support of the conclusion that the two factors act on antibacterial activities independently and are mechanistically unrelated. In both cases, however, the measurements of the same species were close together on the dendrograms (Fig. S3A and C).

10.1128/mBio.01172-17.4 FIG S3  PCA and clustering of efflux and OM ratios in different species. (A) Heat map of efflux ratios measured in different species. Efflux ratios (WT/Efflux mutant) with native membranes (Bt, *A. baumannii* ATCC 17978, PAO1, and *E. coli*) and those of hyperporinated strains (Bt-Pore, Abau-Pore, PAO1-Pore and *E. coli*-Pore) are shown. The scale represents log_2_(fold change in MICs). (B) PCA of efflux ratios. Clusters are numbered as described for panel A. (C) A heat map of OM ratios (parent/parent-Pore) for efflux-proficient strains (Bt, *B. cepacia* ATCC 25416, *P. aeruginosa*, PAO1, and *E. coli*) and efflux-deficient strains (AbΔ3, PΔ3, ΔTolC, and BtΔ2) is shown. The scale represents log_2_(fold change in MICs). The clusters of antibiotics are numbered. (D) PCA of OM ratios. Clusters are numbered as described for panel C. Download FIG S3, TIF file, 1.2 MB. Copyright © 2017 Krishnamoorthy et al. 2017 Krishnamoorthy et al. This content is distributed under the terms of the Creative Commons Attribution 4.0 International license.

Notably, the found clusters were broadly distributed across the physicochemical space of the tested antibiotics (Fig. 5C). This further supports the conjecture that biology, not chemistry, governs compound permeation into cells. The distribution of antibiotics from each cluster appeared to follow its own pattern and to converge into the same quadrant of the property plot, which is occupied by relatively hydrophobic compounds with a MW below 600. The physicochemical determinants of cell entry in the studied species remain unknown. However, branching of clusters into divergent physical spaces is driven by biological determinants such as the requirement for specific porins in the OM (group 1), targeting of the OM (group 3), or specific recognition by efflux pumps (group 2) (Fig. 5B).

## DISCUSSION

In this study, we analyzed the contributions of the OM permeability and active efflux to the intracellular accumulation and antibacterial activities of four divergent bacterial species. We found that *P. aeruginosa* PAO1, *A. baumannii* ATCC 17978, Bt, and *B. cepacia* ATCC 25416 differ significantly from each other in the specificities and capacities of their active efflux pumps and in how these pumps interact with the OM barriers. As seen from the increased accumulation of fluorescent probes (Fig. 2 and 3), hyperporination of the OM was successful in all four species and increased by severalfold the flux of compounds across the OM. The kinetic studies of intracellular accumulation of fluorescent probes showed that hyperporination and active efflux act on the permeation of compounds independently from each other but in a species-specific manner. Further analyses demonstrated the lack of interdependence of the permeability barriers in antibacterial activities as well.

First, in agreement with results of intracellular accumulation experiments, the effects of efflux and OM on antibacterial activities cluster separately, indicating that these two barriers affect antibiotics independently from each other and hence are mechanistically unrelated (Fig. 5A). Second, all antibiotics are affected by permeability barriers and form four distinct clusters (Fig. 5B), suggesting that, despite an apparent dominance of biological determinants, antibiotics within the clusters share structural features that are recognized by different barriers. Finally, antibiotics from the same clusters relate to each other through their interactions with permeability barriers and occupy broad but defined physicochemical spaces (Fig. 5C). The existing heuristics emphasize the size and polarity of compounds as major determinants of permeation across the OM and the propensity to be recognized by efflux pumps. In general, very polar and low-MW compounds and zwitterionic and high-MW compounds are thought to avoid efflux, whereas the hydrophobicity of compounds positively correlates with efflux ratios (8, 21, 22). The four clusters of antibiotics identified here do not conform to these rules (Fig. 5C). It should be recognized that, despite the chemical diversity and apparent lack of chemical similarity of the antibiotics within the clusters, the biological recognition is based on similar properties; i.e., substrates share certain properties if they are recognized by the same efflux pump. Hence, further analyses of such properties should uncover rules of permeation specific to identified clusters of antibiotics.

The mechanistic separation between efflux and OM effects is apparent despite significant interspecies differences in the structures of OMs and in the specificities of efflux pumps. The overall structural organizations of all Gram-negative OMs are thought to be similar and comprise an asymmetric bilayer of lipopolysaccharide (LPS) phospholipids with general and specific porins and channels embedded in it (13, 47). Undoubtedly, structural features and the variability of both lipids and proteins of OMs contribute to antibiotic permeation. *P. aeruginosa* PAO1 lipid A is structurally the closest to that of *E. coli* (Fig. 1) and contains the 10-to-12-carbon-long acyl chains that create a hydrophobic layer 18 Å in thickness. The OM of *P. aeruginosa* PAO1, however, does not contain large nonspecific porins comparable to OmpF of *E. coli*, the factor limiting significantly the uptake of hydrophilic antibiotics (Table 1) (48, 49). Consistent with the lack of general porins, with a few exceptions, hyperporination significantly potentiated antibacterial activities in *P. aeruginosa* PAO1 (see Fig. S2 and S5A in the supplemental material). Lipid A of *A. baumannii* ATCC 17978 is hexa- and hepta-acylated with fatty acids of 12 and 14 carbons in length (50, 51). As a result, the hydrophobic core of *A. baumannii* ATCC 17978 is expected to be thicker (~23 Å) and lipid A is expected to occupy a larger area per lipid (Fig. 1). These features are likely to make the OM of *A. baumannii* ATCC 17978 more hydrophobic and are responsible for the modest effect of *A. baumannii* ATCC 17978 hyperporination for most antibiotics and the hypersusceptibility of this species to such amphiphilic antibiotics as macrolides, novobiocin, and tetracycline, the antibiotics to which other Gram-negative species are resistant (Table 1, Table 2, and Table 3). *Burkholderia* spp. contain tetra- and penta-acylated lipid A with the longest acyl chains of 14 and 16 carbons and an expected thickness of ~24 to 25 Å (52, 53). The structures of lipid A in *B. cepacia* ATCC 25416 and Bt are very similar and contain a characteristic 4-amino-4-deoxy-l-arabinose (Ara4N) substitution linked to one or both phosphate groups in the lipid A backbone and in the core region of LPS (Fig. 1). In other Gram-negative species, Ara4N modifications are present during infections as a protection against antimicrobial peptides (54–57). We found that, in addition to other antibiotics, these modifications enable the synergism with active efflux against aminoglycosides and polymyxin, antibiotics that are otherwise privileged in their permeation across Gram-negative cell envelopes (Table 2). These structurally diverse OMs present a wide diversity of possible interactions. Yet the molecular determinants of antibiotic interactions with OM are specific and different from those of efflux pumps.

RND pumps are the major contributors to intrinsic and clinical antibiotic resistance in Gram-negative bacteria and are effective against a broad range of structurally unrelated compounds (17). We unexpectedly found that the functions of RND pumps universally affect activities of all tested antibiotics, even those previously considered to be outside the efflux recognition space (22, 58). The effect of efflux on large antibiotics could not be assessed before, because such antibiotics as zeocin, bacitracin, and vancomycin penetrate the OM too slowly and do not accumulate significantly in cells. However, hyperporination enabled penetration of these large antibiotics into periplasm and their active efflux. Our results demonstrate that the chemical space affected by active efflux is much broader than previously thought and that the size of a compound is not a characteristic predictive of an efflux substrate. This result has further implications for the mechanism of efflux pumps, as the substrate binding sites and tunnels of transporters (59, 60) must be able to accommodate substrates that are significantly larger than previously thought.

There are also significant species-specific efflux effects. The efflux contribution in *A. baumannii* ATCC 17978 is largely defined by the activity of AdeIJK, the major constitutively expressed efflux pump of this species (61). Although the strains used in this study are susceptible to most of the tested antibiotics, *A. baumannii* ATCC 17978 is notorious for rapid development of multidrug resistance in clinical settings. Overproduction of the AdeABC efflux system is observed with a high incidence in multidrug-resistant *A. baumannii* ATCC 17978 isolates and results in increased resistance to several antibiotics of choice for the treatment of infections caused by this nosocomial pathogen (20). Our results predict that, in order to compensate for the “leaky” OM of *A. baumannii* ATCC 17978 and provide antibiotic resistance, the overexpression of these pumps must be very efficient. MexAB-OprM is the major efflux pump of *P. aeruginosa* PAO1, shows broad substrate specificity (34), and is likely to be responsible for the observed efflux effects (Table 1 and Fig. S2). MexCD-OprJ and MexXY-OprM, also lacking in the PΔ3 strain, are commonly overproduced in clinical isolates (18, 62). While inactivation of efflux made *P. aeruginosa* PAO1 hypersusceptible to various antibiotics, the OM of strain PΔ3 became less permeable to fluorescent probes, as seen from the reduced initial rates of HT uptake (Fig. 2B). Thus, *P. aeruginosa* PAO1 cells compensate for the loss of efflux pumps by further reducing the permeability of the OM.

*B. cepacia* ATCC 25416 and Bt differ dramatically from each other and other species in their efflux capacities. Our results further suggest that even the limited (3 to 4 pores per cell) (Fig. 1) hyperporination of Bc-RHA-BtPore increased the influx of antibiotics sufficiently to overwhelm the *B. cepacia* ATCC 25416 efflux pumps. As a result, these cells were hypersusceptible to all tested antibiotics. In contrast, because of highly effective efflux, the influx remained very slow in the hyperporinated Bt-RHA-BtPore cells. Indeed, hyperporination had a dramatic effect in BtΔ2-RHA-BtPore cells lacking efflux pumps (Table 2), splitting the two hyperporinated Bt strains into two distant clusters (Fig. 5A). In Bt cells, efflux inactivation alone was responsible for potentiation of the antibacterial activities of antibiotics in all four clusters, suggesting that efflux pumps are the dominant contributors to the permeability barrier of this species. Like *P. aeruginosa* PAO1, *Burkholderia* spp. contain a large array (10 to 16 operons, depending on the species) of RND efflux pumps (63, 64). Among these pumps, only three have been characterized in detail in Bt (35) and none in *B. cepacia* ATCC 25416 but several transporters are known to function in *B. cenocepacia* (64, 65). AmrAB-OprA of Bt is related to MexXY-OprM of *P. aeruginosa* PAO1 and contributes to resistance to macrolides and aminoglycosides (35). BpeAB-OprB is a broad-specificity pump responsible for intrinsic antibiotic resistance (66). It was only in Bt cells that inactivation of efflux potentiated activities of aminoglycosides by up to 128-fold independently in the presence or absence of the pore. In contrast, the synergism between inactivation of efflux and hyperporination in BtΔ2-RHA-BtPore was strong with respect to the activities of all other antibiotics, including polymyxin B. These results suggest that efflux-deficient Bt cells present to aminoglycosides and polymyxin B an OM that is susceptible to the action of these polycationic antibiotics. Hence, the Bt efflux pumps are needed to maintain the proper structure of the OM and their inactivation could lead to modifications of LPS. As a result of these structural changes in OM, BtΔ2-RHA-BtPore cells became even more susceptible to antibiotics than the cells of the analogous *P. aeruginosa* PAO1 and *A. baumannii* ATCC 17978 strains. Thus, dramatic differences in the intrinsic levels of antibiotic resistance among the species could be attributed almost entirely to the differences in permeability barriers of their cell envelopes.

The strains and assays developed in this study are expected to facilitate medicinal chemistry efforts to improve activities of compounds and broaden their antibacterial spectra. In particular, the “barrier-less” strains allow access to conserved and species-specific intracellular targets, whereas hyperporinated and efflux-deficient variants separate the contributions of the two barriers in antibacterial activities and improve access to different intracellular compartments. Since *P. aeruginosa* or enterobacteria augment their LPS with the same chemical groups that are constitutive in Bt and *B. cepacia*, a combination of strains with OMs of different compositions could be used to predict the effects of LPS modifications on antibiotic activities during infections.

## MATERIALS AND METHODS

The construction of bacterial plasmids and strains is described in Table S1 in the supplemental material. Primers used in the construction of plasmids are listed in Table S2.

10.1128/mBio.01172-17.7 TABLE S2  Primers used in this study. Download TABLE S2, DOCX file, 0.01 MB. Copyright © 2017 Krishnamoorthy et al. 2017 Krishnamoorthy et al. This content is distributed under the terms of the Creative Commons Attribution 4.0 International license.

Luria-Bertani (LB) broth (10 g of Bacto tryptone, 5 g of yeast extract, and 5 g of NaCl per liter; pH 7.0) or LB agar (LB broth with 15 g of agar per liter) was used for bacterial growth. When indicated, cultures were induced with 0.1 mM isopropyl β-d-1-thiogalactopyranoside (IPTG), 1% l-arabinose, or 0.2% l-rhamnose to induce expression of “Pore” proteins. For selection, gentamicin (for *P. aeruginosa*, 30 µg/ml or 15 µg/ml; for *A. baumannii*, 30 µg/ml), trimethoprim (for *B. thailandensis*, *B. cepacia*, *E. coli*, and *A. baumannii*, 100 µg/ml), tellurite (for *E. coli*, 10 µg/ml), ampicillin (100 µg/ml), carbenicillin (200 µg/ml), streptomycin (100 µg/ml), and polymyxin B (10 µg/ml or 25 µg/ml) were used. Susceptibility to different classes of antibiotics was determined by a 2-fold broth dilution method (25).

Uptake assay was performed in a temperature-controlled microplate reader (Tecan Spark 10M multimode microplate reader equipped with a sample injector) in fluorescence mode (24). Cells from frozen stocks were inoculated into LB medium and incubated for 16 h at 37°C. Cells were then subcultured into a fresh 30-ml volume of LB medium and grown at 37°C to an optical density at 600 nm (OD_600_) of 0.3. The cells were then induced with 0.1 mM IPTG or 0.2% rhamnose or 1% arabinose and grown to an OD_600_ of 1.0, collected by centrifugation at 4,000 rpm for 40 min at room temperature, and washed in 25 ml HEPES-KOH buffer (50 mM; pH 7.0) containing 1 mM magnesium sulfate and 0.4 mM glucose (HMG buffer). The cells in HMG buffer were adjusted to an OD of ~1.0 and kept at room temperature during the experiment. Fluorescence intensities from HT and NPN uptake experiments were plotted against time in Microsoft Excel and normalized to the emission before cells were added. The data were imported into MatLab (MathWorks, Inc.) to be fitted to a simple exponential equation in the form of *F* = *A*_1_ + *A*_2_[1 − exp(−kt)], where *A*_1_ represents the amplitude of the initial fast uptake of HT, and *A*_2_ and kt are the amplitude and rate, respectively, that are associated with the subsequent slower uptake (24).

For protein analyses, membrane fractions were isolated from *P. aeruginosa* or Bt or *B. cepacia* cells by ultracentrifugation as described before (25). Outer membrane fractions were enriched by solubilization of inner membrane proteins in buffer containing 50 mM Tris-HCl (pH 8.0), 150 mM NaCl, 1 mM phenylmethylsulfonyl fluoride (PMSF), and 0.2% Triton X-100 and separated from the insoluble outer membrane fractions by ultracentrifugation. The resulting pellet was further solubilized in 50 mM Tris-HCl (pH 8.0), 150 mM NaCl, 1 mM PMSF, and 5.0% Triton X-100 followed by ultracentrifugation to remove insoluble components. The supernatant was incubated with His•Bind resin (Novagen), which was previously charged with 50 mM CuSO_4_. The pore protein was eluted with 20 mM Tris-HCl (pH 8.0), 500 mM NaCl, 1 mM PMSF, 0.2% Triton X-100, and 400 mM imidazole. The eluted fractions were analyzed by 12% SDS-PAGE followed by immunoblotting with primary monoclonal anti-histidine tag antibodies (Thermo Fisher Inc.) and a secondary alkaline phosphatase-conjugated anti-mouse antibody (Sigma). The 5-bromo4-chloro-3-indoyl phosphate (BCIP) and nitroblue tetrazolium (NBT) substrates were used to visualize the bands. The pore was quantified using Quantity One software (Bio-Rad) with His-tagged *P. aeruginosa* protein TriC as a standard.

Hierarchical clustering was done using the MatLab Statistics toolbox using unweighted average Euclidean distances between logarithms of measured MIC ratios. Principal-component analysis (PCA) revealed that the first two components described the effect of hyperporination and active efflux and were responsible for 77% of the overall variance and for 93% of the variance for four components (Table S3). The coordinates of the PCA vectors are shown in Table S3 together with the percentage of explained variance for each vector. The *R*-squared index analysis (67) suggested the existence of three clusters in the distribution (see Fig. S4 in the supplemental material). PCA coordinates were then fitted to a Gaussian mixture model using an expectation maximization (EM) algorithm. The hierarchical algorithms and EM algorithms produced the same separation of data into clusters, which did not change upon the removal of up to 20% of constituent points from each cluster.

10.1128/mBio.01172-17.8 TABLE S3  Coordinates and the explained variance of the principal components. Download TABLE S3, DOCX file, 0.02 MB. Copyright © 2017 Krishnamoorthy et al. 2017 Krishnamoorthy et al. This content is distributed under the terms of the Creative Commons Attribution 4.0 International license.

10.1128/mBio.01172-17.5 FIG S4  Calculated RMSSTD (root mean square standard deviation) and RS (*R*-squared) internal validation indices. Download FIG S4, TIF file, 2.3 MB. Copyright © 2017 Krishnamoorthy et al. 2017 Krishnamoorthy et al. This content is distributed under the terms of the Creative Commons Attribution 4.0 International license.
