# Supplementary material for: Synergy between Active Efflux and Outer Membrane Diffusion Defines Rules of Antibiotic Permeation into Gram-Negative Bacteria
Source: mBio. 2017 Oct 31;8(5):e01172-17. doi: 10.1128/mBio.01172-17 (PMC5666154; doi:10.1128/mBio.01172-17)
Supplement: TABLE S1 [file mbo005173551st1.docx]

**Supplemental Tables**

Table S1: Strains and Plasmids

| Strain | Relevant Genotype | Source |
| --- | --- | --- |
| *P. aeruginosa* PAO1 | Wild type | O. Lomovskaya |
| GKCW111 | PAO1 *att*Tn*7*::mini-Tn*7*T-Gm-*lacI^q^*-pLAC-MCS | This study |
| GKCW119 | PAO1 *att*Tn*7*::mini-Tn*7*T- Gm^r^-*lacI^q^*-pLAC- *fhuAΔCΔ4L* | This study |
| PAO1 (Pa∆3L) | PAO1 but Δ*mexAB* Δ*mexCD* Δ*mexXY* | O. Lomovskaya |
| GKCW112 | PAO1∆3 *att*Tn*7*::mini-Tn*7*T- Gm^r^-*lacI^q^*-pLAC-MCS | This study |
| GKCW120 | PAO1∆3 *att*Tn*7*::mini-Tn*7*T- Gm^r^-*lacI^q^*-pLAC- *fhuAΔCΔ4L* | This study |
| *B. thailandensis* E264 | Wild type | H. Schweizer |
| Bt-RHA | E264 *glmS1* *att*Tn*7*::miniTn*7*-Tp-RHA | This study |
| Bt-RHA- BtPore | E264 *glmS1* and *glmS2* *att*Tn*7*::miniTn*7*-Tp-RHA-*orbA* ((OrbA lacking the cork and four extracellular loops, and with C-terminal 9His tag). | This study |
| Bt38 (BtΔ2) | E264 Δ*bpeAB-oprA*::FRT Δ*amrAB-oprA*::FRT | H. Schweizer |
| BtΔ2-RHA | Bt38 *glmS1* *att*Tn*7*:: miniTn*7*-Tp-RHA | This study |
| BtΔ2-RHA-BtPore | Bt38 *glmS1* and *glmS2* *att*Tn*7*::miniTn*7*-Tp-RHA-*orbA* | This study |
| *B. cepacia* 25416 | Wild type | ATCC |
| Bc-RHA | Bc *att*Tn*7*::miniTn*7*-Tp-RHA | This study |
| Bc-RHA- BtPore | Bc *att*Tn*7*::miniTn*7*-Tp-RHA-*orbA* (OrbA_ΔCork, Δ4Loop, 9His_); expresses OrbA | This study |
| *A. baumanii 17978* | Wild type | ATCC |
| JWW19 (Ab-ARA) | ATCC 17978 *att*Tn*7*::miniTn*7*-Tp^r^-*araC*-P*_araBAD_-*MCS (pTJ1) | This study |
| JWW20 (Ab-ARA-EcPore) | ATCC 17978 *att*Tn*7*::miniTn*7*-Tp^r^-*araC*-P*_araBAD_* -*fhuA* (FhuA_ΔCork, Δ4Loop, 6His_) (pTJ1-FhuA) | This study |
| IL119 (AbΔ3) | ATCC 17978 Δ*adeIJK*::FRT Δ*adeAB*::FRT Δ*adeFGH*::FRT | This study |
| IL122 (AbΔ3-ARA) | ATCC 17978 Δ3 *att*Tn*7*::miniTn*7*-Tp^r^-*araC*-P*_araBAD_*-MCS (pTJ1) | This study |
| IL123 (AbΔ3-ARA-EcPore) | ATCC 17978 Δ3 *att*Tn*7*::miniTn*7*-Tp^r^-*araC*-P*_araBAD_*-*fhuA*(FhuA_ΔCorkΔ4Loop, 6His_) (pTJ1-FhuA) | This study |
| *Plasmids* |  |  |
| pTNS3^a, b^ | Ap^r^; Helper plasmid encoding Tn*7* transposase proteins TnsABCD from P1 and P*_lac_* promoter | (1) |
| pUC18T-mini-Tn7T-LAC- (Gm^r^) ^a, b^ | A suicide delivery vector | (1) |
| pUC18T-mini-Tn7T-*araC-P_BAD_*-(Tp^r^)^a, b^ | A suicide delivery vector | (1) |
| pGK-LAC-FhuA ∆C/∆4L (Gm^r^) ^a, b^ | pUC18T mini-Tn*7*T- *LAC-* Gm^r^-vector carrying *fhuA ∆C/∆4L* gene | (2) |
| pDW- *araC*-P*_araBAD_*-*fhuA* ∆C/∆4L -(Tp^r^)  ^a, b^ | pUC18T mini-Tn*7*T- *araC-P_BAD_-* Tp^r^ vector carrying f*huA ∆C/∆4L* gene | (2) |
| pPR-IBA1- FhuA ∆C/∆4L | pET-based plasmid containing f*huA ∆C/∆4L* gene | (3) |
| pUC18T-mini-Tn7-Tp-RHA^b^ | Ap^r^; Tp^r^; miniTn7T cassette containing *rhaRS*-P*_rhaBAD_* | This study |
| pUC18T-mini-Tn7-Tp-RHA-*orbA* | pUC18T mini-Tn*7*T- *LAC-* Tp^r^-vector carrying OrbA_ΔCork, Δ4Loop, 9His_ from P*_rhaBAD_* | This study |
| pAT02 | pMMB67EH with Rec_Ab_ system, Amp^r^ | (4) |
| pAT03 | pMMB67EH with FLP recombinase, Amp^r^ | (4) |
| pMo130-TelR ^a^ | Suicide plasmid, *xylE^+^, sacB^+^,* Km^r^, Tel^r^ | (5) |
| pIL117 | pMo130-Tel^r^ plasmid containing gentamicin-resistance cassette, Gm^r^ | This study |
| pIL118 | pMoT*∆adeAB*::Gm^r^ containing 0.5 kb UP (*adeA*) and 0.5 kb DOWN (*adeB*) fragments; Tel^r^, Gm^r^ | This study |
| pIL119 | pMoT*∆adeFGH*::Gm^r^ containing 0.5 kb UP (*adeF*) and 0.5 kb DOWN (*adeH*) fragments; Tel^r^, Gm^r^ | This study |
| pIL121 | pMoT*∆adeIJK*::Gm^r^ containing 1 kb UP (*adeI*) and 1 kb DOWN (*adeK*) fragments; Tel^r^, Gm^r^ | This study |
| pEx18Ap | *oriT*^+^ *sacB*^+^ gene replacement vector with multiple-cloning site from pUC18; Amp^r^ | H. Schweizer |
| pIL127 | pEx18Ap ∆adeIJK::Gm containing 1 kb UP (*adeI*) and 1 kb DOWN (*adeK*) fragments; Amp^r^, Gm^r^ | This study |
| pTJ1^b^ | Ap^r^; Tp^r^; miniTn7T cassette containing *araC*-P*_araBAD_* | (6) |

^a^Ap^r^, Gm^r^, Tel^r^, Tp^r^ genes encoding resistance to ampicillin, gentamicin, tellurite, and trimethoprim, respectively**.**

^b^P*_lac_*, P*_araBAD_*, P_TAC_, P*_rhaBAD_*, encode the *E. coli* lac, arabinose, lac/trp hybrid, and rhamnose promoters; P1 encodes the P1 integron promoter; P*_S12_* encodes the *B. thailandensis* ribosomal protein S12 promoter; PC_S12_ encodes the *B. cenocepacia rpsL* promoter driving transcription of

Tel^r^ or Tp^r^ genes; P_λ_ encodes the λ repressor promoter

**References**

1. Choi K-H, Schweizer HP. 2006. mini-Tn7 insertion in bacteria with single attTn7 sites: example Pseudomonas aeruginosa. Nat Protocols 1:153-161.

2. Krishnamoorthy G, Wolloscheck D, Weeks JW, Croft C, Rybenkov VV, Zgurskaya HI. 2016. Breaking the Permeability Barrier of Escherichia coli by Controlled Hyperporination of the Outer Membrane. Antimicrob Agents Chemother 60:7372-7381.

3. Mohammad MM, Howard KR, Movileanu L. 2011. Redesign of a plugged beta-barrel membrane protein. J Biol Chem 286:8000-13.

4. Tucker AT, Nowicki EM, Boll JM, Knauf GA, Burdis NC, Trent MS, Davies BW. 2014. Defining Gene-Phenotype Relationships in Acinetobacter baumannii through One-Step Chromosomal Gene Inactivation. mBio 5.

5. Amin IM, Richmond GE, Sen P, Koh TH, Piddock LJ, Chua KL. 2013. A method for generating marker-less gene deletions in multidrug-resistant Acinetobacter baumannii. BMC Microbiol 13:158.

6. Damron FH, McKenney ES, Barbier M, Liechti GW, Schweizer HP, Goldberg JB. 2013. Construction of mobilizable mini-Tn7 vectors for bioluminescent detection of gram-negative bacteria and single-copy promoter lux reporter analysis. Appl Environ Microbiol 79:4149-53.
