# Supplementary material for: Synergy between Active Efflux and Outer Membrane Diffusion Defines Rules of Antibiotic Permeation into Gram-Negative Bacteria
Source: mBio. 2017 Oct 31;8(5):e01172-17. doi: 10.1128/mBio.01172-17 (PMC5666154; doi:10.1128/mBio.01172-17)
Supplement: TEXT S1 [file mbo005173551s1.docx]

**Synergy between active efflux and outer membrane diffusion defines rules of antibiotic permeation into Gram-negative bacteria**

Ganesh Krishnamoorthy*, Inga Leus*, Jon W. Weeks*, David Wolloscheck, Valentin V. Rybenkov and Helen I. Zgurskaya^1^

Department of Chemistry and Biochemistry, University of Oklahoma, Norman, OK73019

**Supplemental Methods**

*Construction of plasmids and strains used in this study*

***P. aeruginosa (Pae) strains.*** *To* construct pGK-LAC-fhuA ∆C/∆4L (Gm^r^), the gene encoding FhuA ∆C/∆4L (EcPore) was amplified from the pPR-IBA1-FhuA ∆C/∆4L plasmid. The PCR product was ligated with the pUC18-mini-Tn7T-LAC suicide delivery vector restricted with SacI and KpnI enzymes and transformed into *E. coli* DH5α competent cells and plated on LB agar plates containing Gentamycin (30 µg/ml). The colonies were screened for the insertion of FhuA ∆C/∆4L gene into the vector by restriction digest analysis.

Insertion of mini-Tn7T-LAC-FhuA ∆C/∆4L (Gm^r^) onto the chromosome of *P. aeruginosa* PAO1 strain was achieved as described by Choi and Schweizer(1). Briefly, the suicide delivery vector carrying the *fhuA ∆C/∆4L* gene and the helper plasmid PTNS3 were electroporated into PAO1 and Pa∆3 strains and grown for 1 h in LB medium containing 1 mM glucose. The cells were then plated onto LB agar containing gentamicin at 30 µg/ml (PAO1) or 15 µg/ml (Pa∆3) and incubated for 16h at 37^o^C. Resulting colonies were selected and confirmed for the insertion by PCR using glmS down and glmS UP primers (Table S2).

***B. thailandesis (Bt) and B. cepacia (Bc) strains.*** The *B. thailandensis* *orbA* gene encodes a homologue of the *E. coli* FhuA siderophore receptor protein. The gene encoding *orbA* was modified similarly to the synthetic *fhuAΔC/Δ4L* gene(2), in which the periplasmic cork domain and the extracellular loops of OrbA protein have been removed. This synthetic *orbA* (OrbA ∆C/∆4L) gene was synthesized by GenScript Corporation and cloned into pUC57 between the SacI/HindIII sites. A NcoI site was engineered to encode the Start Codon for cloning into pUC18T-mini-Tn7T-RHA, which encode optimized Shine-Dalgarno sequences in frame to their NcoI sites. To construct pUC18T-mini-Tn7T-Tp-RHA, the *rhaRS*-P*_rhaBAD_* promoter was amplified with primers RhaR NsiI REV and P-rhaBAD SpeI/NcoI REV (Table S2). PCR product and pUC18T-miniTn7T-Tp were digested with NsiI/SpeI and ligated using T4 ligase, following manufacturer’s protocols (NEB).

Mini Tn*7* cassettes were routinely integrated onto the chromosome of *B. thailandensis* by tri-parental mating with recipient *B. thailandensis*, RHO3/pTNS3, and SM10λ*pir*+ carrying pUC18T-based mini Tn*7* cassettes. Mating was carried out by growing strains on LBA plates with appropriate selection markers. Overnight cultures were diluted, 1 to 100 for recipient and RHO3/pTNS3 or 1:20 for SM10λ*pir*+ carrying pUC18T-based mini Tn*7* cassettes, into fresh LB containing appropriate selection markers and grown at 37°C to OD ~0.2. Cells were collected by centrifugation at 3220 x g for 20 min and gently resuspended in LB to concentrate 100x. 10 µl of each strain was mixed in a sterile centrifuge tube and 10 µl of tri-parental mixture was spotted and allowed to adsorb onto a dried LBA plate containing 0.3 mM diamino palmitic acid. Mating reactions were incubated at 37°C for 16 hours. Spots were scraped off the LBA plates and resuspended in 500 µl of LB. 100 µl of resuspended mating reaction was plated onto LBA containing 25 µg/ml polymyxin B and 100 µg/ml trimethoprim. Integrants were screened by stable growth on LBA plates containing 25 µg/ml polymyxin B and 100 µg/ml trimethoprim for Bt and 10 µg/ml polymyxin B and 10 µg/ml trimethoprim, pigment production on LBA for B*. cepacia*, as well as PCR confirmation of integration into either or both *att*Tn*7* sites at *glmS1* or *glmS2*.

***A. baumannii (Ab).*** A suicide vector harboring a tellurite-resistance marker pMo130-Tel^R^ was first created by inserting a gentamicin-resistance cassette to replace a kanamycin-resistance marker. To construct the suicide plasmid for deletion of efflux pumps, a 1 kb (for *adeIJK*) and 0.5 kb (for *adeFGH* and *adeAB*) DNA fragment located upstream and downstream of the efflux pump operons were amplified from genomic DNA. The constructed pIL118, pIL119 and pIL121 plasmids were used as templates to amplify ∆adeIJK::Gm, ∆adeFGH::Gm and ∆adeAB::Gm fragments, correspondingly.

The inactivation of *adeIJK* gene was done by cloning ∆adeIJK::Gm PCR product into pEX18Ap. The resulting plasmid construct pIL127 was first introduced into *E. coli* SM10λpir and subsequently delivered into *A. baumannii* by biparental conjugation. *A. baumannii* transconjugants (first crossovers) were selected on LB agar containing 30 µg/ml gentamicin and 100 µg/ml trimethoprim. During the second cross-over, mutants with gene deletion were selected for loss of *sacB* by passaging the first cross-over recombinants in LB agar containing 10% sucrose.

The inactivation of *adeAB* and *adeFGH* genes were done using RecAB as described(3). We used electrocompetent cells at a density of 10^10^ CFU/reaction and 5 µg of PCR product with 0.5 kb of flanking homology. We obtained approximately 10-50 colonies from each transformation, depending on the gene being targeted for replacement. The gentamicin resistance cassette was removed by transformation of the pAT03 plasmid and activation of the FLP recombinase enzyme. Genomic deletions of the *adeAB*, *adeFGH* and *adeIJK* operons in the mutants were verified by comparing the PCR amplimers obtained from the wild type strain and corresponding pump gene deletion mutants.

Mini Tn*7* cassettes were routinely integrated onto the chromosome of *A. baumannii* WT and Ab∆3 strains by tri-parental mating with recipient *A. baumannii*, RHO3/pTNS3, and SM10λ*pir*+ carrying pTJ1 or pDW- *araC*-P*_araBAD_*-*fhuA* ∆C/∆4L (Table S1). Mating was carried out by growing strains on LBA plates with appropriate selection markers. Overnight cultures were diluted, 1 to 100 for recipient and RHO3/pTNS3 or 1:20 for SM10λ*pir*+ carrying pDW- *araC*-P*_araBAD_*-*fhuA* ∆C/∆4L, into fresh LB containing appropriate selection markers and grown at 37°C to OD ~0.5. Cells were collected by centrifugation at 4000 x g for 20 min and gently resuspended in LB to concentrate 100x. 10 µl of each strain was mixed in a sterile centrifuge tube and 10 µl of tri-parental mixture was spotted and allowed to adsorb onto a dried LBA plate containing 0.3 mM diaminopimelic acid. Mating reactions were incubated at 37°C for 16 hours. Spots were scraped off the LBA plates and resuspended in 500 µl of LB. 100 µl of resuspended mating reaction was plated onto LBA containing 100 µg/ml streptomycin and 100 µg/ml trimethoprim. Integrants were screened by stable growth on LBA plates containing 100 µg/ml streptomycin and 100 µg/ml trimethoprim. Resulting colonies were selected and confirmed for the insertion of EcPore by PCR. We also found that pTJ1-based plasmids used for the chromosomal integration in this and previous studies can self-replicate in this Abau strain (data not shown). This plasmid is also the reason for the β-lactam resistance of the constructed Abau strains (Table 3).

**References**

1. **Choi K-H, Schweizer HP.** 2006. mini-Tn7 insertion in bacteria with single attTn7 sites: example Pseudomonas aeruginosa. Nat Protocols **1:**153-161.

2. **Mohammad MM, Howard KR, Movileanu L.** 2011. Redesign of a plugged beta-barrel membrane protein. J Biol Chem **286:**8000-8013.

3. **Tucker AT, Nowicki EM, Boll JM, Knauf GA, Burdis NC, Trent MS, Davies BW.** 2014. Defining Gene-Phenotype Relationships in Acinetobacter baumannii through One-Step Chromosomal Gene Inactivation. mBio **5**.
