# Supplementary figures and images for: Synergy between Active Efflux and Outer Membrane Diffusion Defines Rules of Antibiotic Permeation into Gram-Negative Bacteria
Source: mBio. 2017 Oct 31;8(5):e01172-17. doi: 10.1128/mBio.01172-17 (PMC5666154; doi:10.1128/mBio.01172-17)

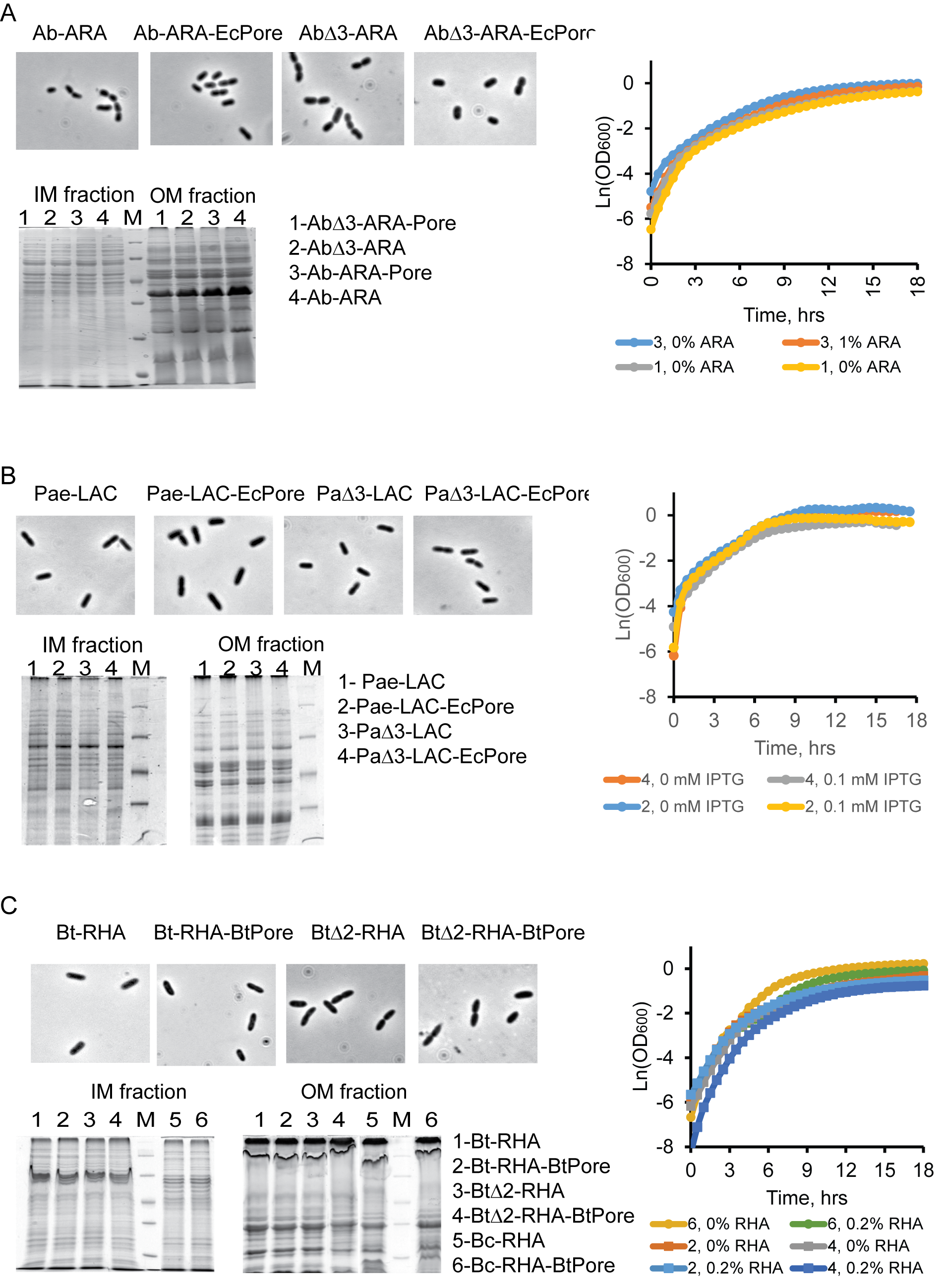

Supplement: FIG S1 [file mbo005173551sf1.tif]

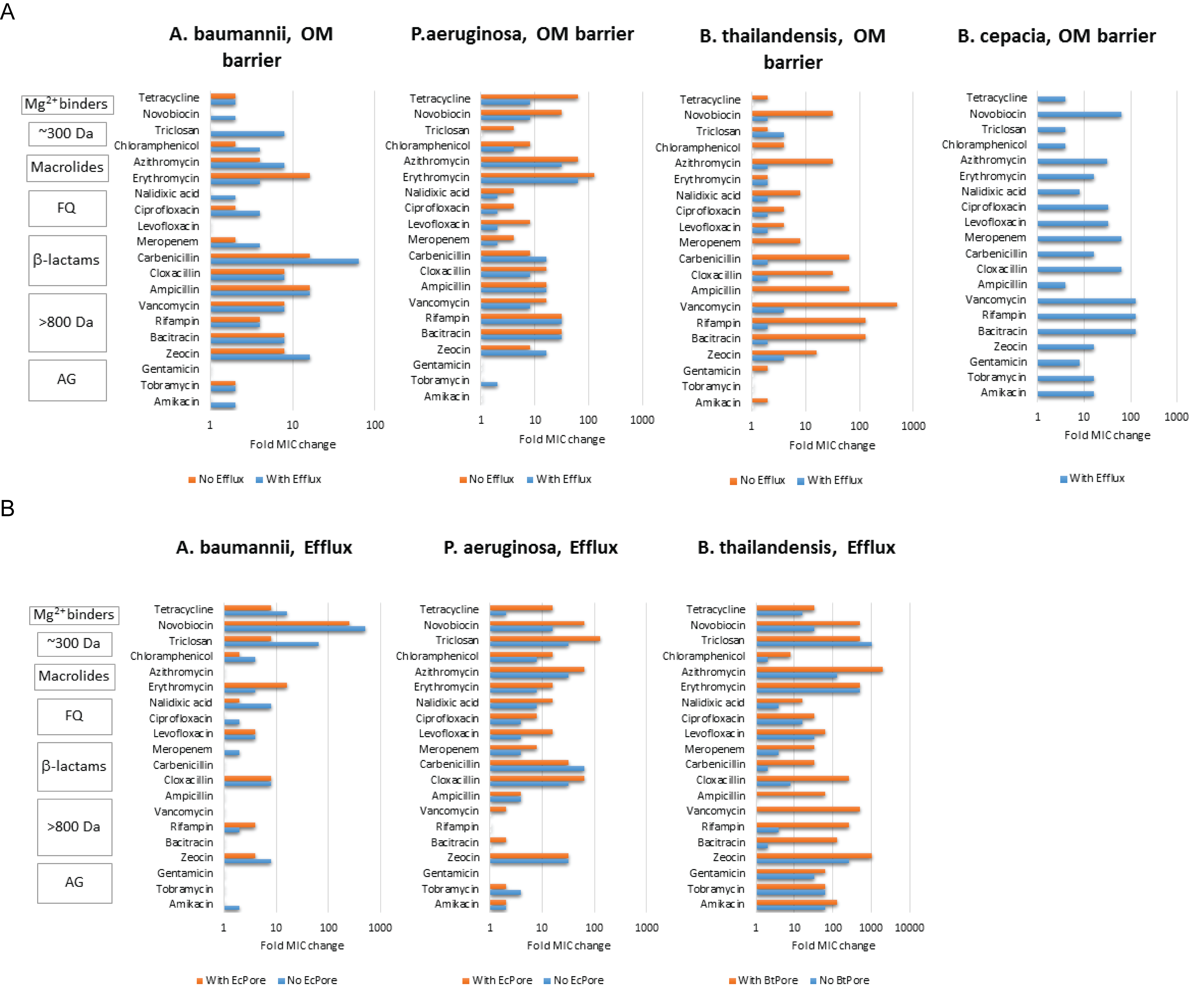

Supplement: FIG S2 [file mbo005173551sf2.tif]

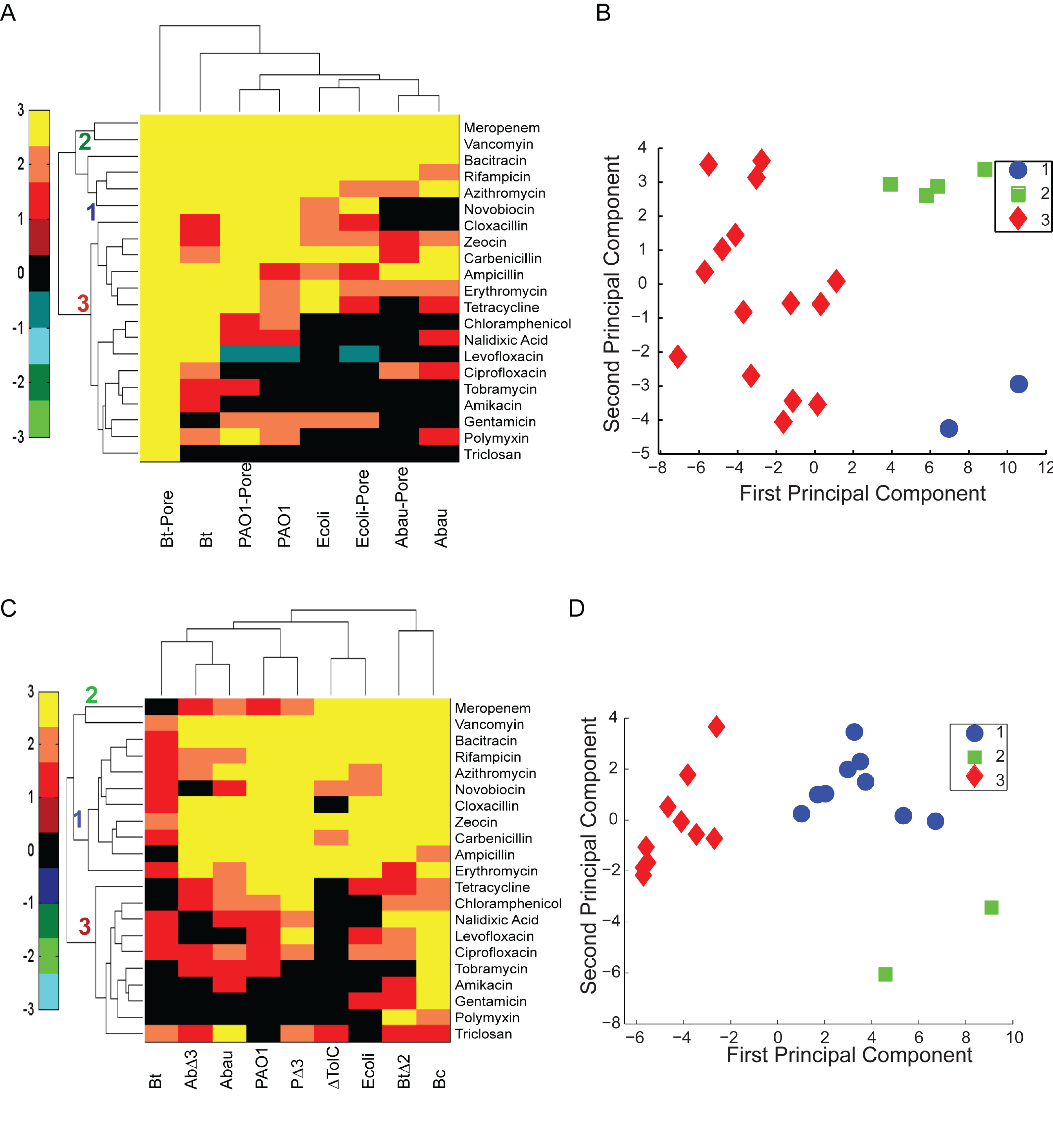

Supplement: FIG S3 [file mbo005173551sf3.tif]

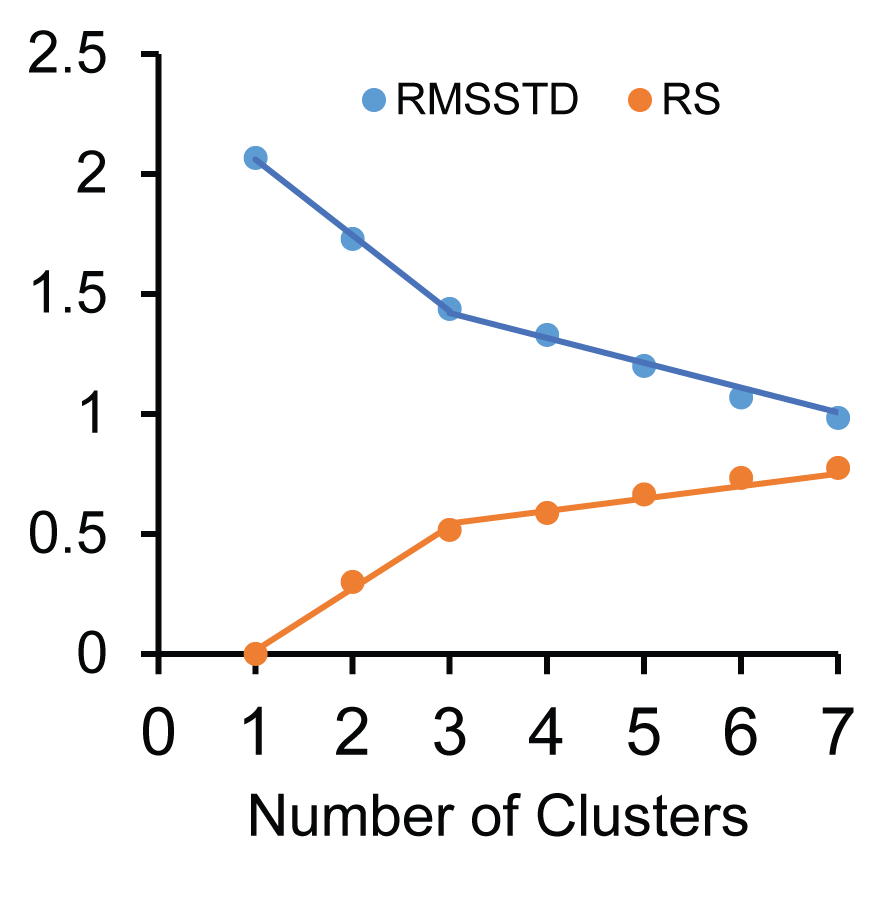

Supplement: FIG S4 [file mbo005173551sf4.tif]
