# Supplementary material for: Synergy between Active Efflux and Outer Membrane Diffusion Defines Rules of Antibiotic Permeation into Gram-Negative Bacteria
Source: mBio. 2017 Oct 31;8(5):e01172-17. doi: 10.1128/mBio.01172-17 (PMC5666154; doi:10.1128/mBio.01172-17)
Supplement: TABLE S2 [file mbo005173551st2.docx]

Table S2: Primers used in this study.

| Primer Name | Sequence 5’ 🡪 3’ |
| --- | --- |
| pH MCS R2 NotI-R | CTAGGCGGCCGCAAGCTTGCATGCCTGCAG |
| RhaR NsiI REV | ATATatgcatTTAATCTTTCTGCGAATTGAGATGACGC |
| P-rhaBAD SpeI/NcoI REV | atatACTAGTccatggTGTGATCCTGCTGAATTTCATTACG |
| SacB-Front BglII | ATATagatctAAAGGATCGATCCTCTAG |
| SacB-End | CAACGTTTGCGCCTAGCTTC |
| pFLP2 x-bla FseI REV | TTAAggccggccCTGTCAGACCAAGTTTACTC |
| pFLP2 x-bla NotI FWD | AATTgcggccgcACTCTTCCTTTTTCAATATTATTGAAGC |
| TelR/TmpR FseI FWD | TTAAggccggccATCCCCTGATTCCCTTTGTC |
| TelR/TmpR NotI REV | TTAAgcggccgcATGTGCTTAAAAACTTACTCAATGG |
| FRT Cass. BamHI FWD (Schweizer) | GTACggatccCGGACGATGAGCTCGAATTGG |
| FRT Cass. BamHI REV (Schweizer) | ATATGGATCCATTAGCTTCAAAAGCG |
| Ab (17978) glmS FWD | TTCGCTGATGAAAATAGTGG |
| Ab (17978) glmS REV | ATTCACCTCAAACCGTACAACG |
| AdeR SphI Int REV | GCTCgcatgcAATTCAATTGCTTGCTTTCC |
| AdeA NsiI Int REV | CGGAATGCATGTACTCGTGCAGGAAG |
| AdeB BamHI Int FWD | atatGGATCCCTGGGTGCAGTAGAAAAGC |
| AdeB Down NotI REV | ATATGCGGCCGCTTAAATACATTTTCGGG |
| AdeF Up SphI FWD | atatGCATGCtttctagtgcaaacacatc |
| AdeF NsiI Int REV | ATATATGCATATTGTGAAAAGTAAATCACC |
| AdeH BamHI Int FWD | atatGGATCCagctcagctgaaagg |
| AdeH Down NotI REV | ATATGCGGCCGCGGCTGAAGTATTCATTGC |
| AdeI Up SphI FWD | atatGCATGCtcaacactggcttgtc |
| AdeI NsiI-L Int REV | AATTatgcatGTTACTGTTATTTAAACTGCC |
| AdeK BamHI-L Int FWD | aattGGATCCctgaatacaacttaagcgc |
| AdeK Down NotI REV | ATATGCGGCCGCTAGATGAGGTGTTTGAGG |
| AdeI Ext FWD | CGTGCACGTCGAGTTCGC |
| AdeK Ext REV | GCGTTTGATGAGTTGTTGGCCG |
| AdeS Int Rev | CGCCATCAATAATTCCC |
| AdeB Ext Check | GGCTTTTAGAGTTTCTCG |
| AdeF Ext Check | CGTCCAATCGATACAGGC |
| PA glmS down | GCACATCGGCGACGTGCTCTC |
| PA glmS UP | CTGTGCGACTGCTGGAGCTGA |
| Ab glmS FWD | TTCGCTGATGAAAATAGTGG |
| Ab glmS REV | ATTCACCTCAAACCGTACAACG |
