# Supplementary material for: Synergy between Active Efflux and Outer Membrane Diffusion Defines Rules of Antibiotic Permeation into Gram-Negative Bacteria
Source: mBio. 2017 Oct 31;8(5):e01172-17. doi: 10.1128/mBio.01172-17 (PMC5666154; doi:10.1128/mBio.01172-17)
Supplement: TABLE S3 [file mbo005173551st3.docx]

| **PCA Component** | **1** | **2** | **3** | **4** | **5** | **6** | **7** | **8** | **9** | **10** | **11** | **12** | **13** |
| --- | --- | --- | --- | --- | --- | --- | --- | --- | --- | --- | --- | --- | --- |
| Explained Variance | 0.390 | 0.250 | 0.125 | 0.074 | 0.056 | 0.038 | 0.034 | 0.015 | 0.010 | 0.004 | 0.002 | 0.001 | 0.000 |
| Cumulative Explained Variance | 0.390 | 0.640 | 0.766 | 0.840 | 0.896 | 0.934 | 0.968 | 0.983 | 0.992 | 0.997 | 0.999 | 1.000 | 1.000 |
| **PCA variable** |  |  |  |  |  |  |  |  |  |  |  |  |  |
| PAO1/ PAO1-Pore | 0.296 | 0.211 | -0.042 | -0.260 | 0.452 | 0.120 | -0.040 | 0.128 | 0.037 | 0.610 | 0.073 | 0.317 | -0.287 |
| PΔ3/  PΔ3-Pore | 0.258 | 0.318 | -0.061 | -0.022 | 0.613 | -0.185 | 0.044 | 0.200 | -0.011 | -0.587 | -0.005 | -0.083 | 0.157 |
| PAO1/ Δ3 | -0.031 | 0.383 | -0.140 | -0.345 | -0.470 | 0.046 | -0.039 | 0.588 | -0.151 | -0.041 | 0.120 | 0.186 | 0.263 |
| BT/BT-Pore | 0.045 | 0.103 | 0.054 | 0.010 | -0.101 | 0.114 | 0.216 | -0.147 | 0.068 | -0.193 | 0.868 | -0.025 | -0.310 |
| BTΔ2/  BTΔ2-Pore | 0.454 | -0.017 | -0.245 | 0.057 | -0.149 | 0.314 | 0.613 | -0.251 | -0.259 | -0.064 | -0.198 | 0.200 | 0.142 |
| BT/BTΔ2 | -0.204 | 0.470 | 0.747 | -0.041 | 0.003 | 0.264 | 0.115 | -0.200 | -0.067 | -0.057 | -0.186 | 0.111 | 0.018 |
| AB/AB-Pore | 0.179 | 0.124 | -0.073 | -0.441 | -0.252 | -0.050 | 0.088 | -0.072 | 0.495 | -0.197 | -0.330 | -0.236 | -0.475 |
| ABΔ3/  ABΔ3-Pore | 0.220 | 0.112 | -0.015 | -0.367 | 0.000 | 0.037 | -0.209 | -0.436 | 0.140 | 0.200 | 0.187 | -0.357 | 0.589 |
| AB/ABΔ3 | -0.143 | 0.376 | -0.102 | 0.356 | -0.053 | -0.466 | 0.431 | -0.005 | 0.415 | 0.299 | 0.011 | 0.010 | 0.182 |
| WT/WT-Pore | 0.443 | 0.004 | 0.154 | 0.216 | -0.241 | -0.231 | -0.416 | -0.181 | 0.227 | -0.171 | 0.020 | 0.576 | 0.052 |
| ΔTolC/  ΔTolC-Pore | 0.480 | -0.025 | 0.383 | 0.101 | -0.178 | -0.395 | 0.055 | 0.193 | -0.389 | 0.190 | 0.013 | -0.419 | -0.145 |
| WT/ΔTolC | -0.040 | 0.553 | -0.403 | 0.304 | -0.101 | 0.076 | -0.364 | -0.278 | -0.321 | 0.024 | -0.090 | -0.180 | -0.263 |
| BC/BC-Pore | 0.257 | 0.019 | 0.071 | 0.450 | -0.030 | 0.576 | -0.116 | 0.362 | 0.396 | 0.058 | -0.009 | -0.285 | 0.089 |

Table S3. Coordinates and the explained variance of the principal components.
